# Nephrotoxicity of Albendazole and Albendazole Loaded Solid Lipid Nanoparticles in Mice with Experimental Hydatidosis

**DOI:** 10.34172/apb.2022.011

**Published:** 2021-07-19

**Authors:** Kobra Kohansal, Abdollah Rafiei, Heibatullah Kalantari, Ali Jelowdar, Anayatollah Salimi, Annahita Rezaie, Mohammad Razi Jalali

**Affiliations:** ^1^Health Research Institute, Infectious and Tropical Diseases Research Center, Ahvaz Jundishapur University of Medical Sciences, Ahvaz, Iran.; ^2^Department of Parasitology, School of Medicine, Ahvaz Jundishapur University of Medical Sciences, Ahvaz, Iran.; ^3^Department of Pharmacology and Toxicology, Faculty of Pharmacy, Toxicology Research Center and Medicinal Plants Research Center, Ahvaz Jundishapur University of Medical Sciences, Ahvaz, Iran.; ^4^Nanotechnology Research Center, Ahvaz Jundishapur University of Medical Sciences, Ahvaz, Iran.; ^5^Department of Pharmaceutics, Faculty of Pharmacy, Ahvaz Jundishapur University of Medical Sciences, Ahvaz, Iran.; ^6^Department of Pathobiology, Faculty of Veterinary Medicine, Shahid Chamran University of Ahvaz, Ahvaz, Iran.; ^7^Department of Clinical Sciences, Faculty of Veterinary Medicine, Shahid Chamran University of Ahvaz, Ahvaz, Iran.

**Keywords:** Albendazole, Nanoparticles, Echinococcus granulosus, Hydatid cyst, Nephrotoxicity

## Abstract

***Purpose:*** Cystic echinococcosis (CE) is a serious contemporary public health problem. Different CE treatment methods are of considerable importance, with albendazole (ABZ) being one of the most preferred drugs for CE treatment and prophylaxis. In this study, we evaluated the nephrotoxicity caused by ABZ and ABZ-loaded solid lipid nanoparticles (SLNs) in mice with experimental hydatid cyst.

***Methods:*** ABZ-loaded SLNs were produced by micro-emulsification and a high shear homogenization technique. Thereafter, we evaluated the physicochemical characterization of the product. Live protoscolices were injected into mice to induce experimental hydatidosis. Mice were then treated with ABZ and ABZ-loaded SLNs. The nephrotoxicity effects were evaluated by biochemical and histopathological surveys.

***Results:*** Significantly different blood urea nitrogen (BUN) levels were observed between the two infected groups (ABZ treatment and ABZ-loaded SLN treatment) and the control group. The kidney malondialdehyde (MDA) and glutathione (GSH) levels of the infected groups were not significantly different from those of the control group. The histopathological study revealed nephropathic and pathologic changes in the ABZ and ABZ-loaded SLN groups.

***Conclusion:*** ABZ formulated for ABZ-loaded SLNs had a more prominent chemoprophylactic efﬁcacy on CE and fewer side effects than ABZ alone. Neither ABZ nor ABZ-loaded SLNs caused significant biochemical and histopathological defects on the kidney, and all functional biochemical markers stayed within the normal range. Therefore, ABZ-loaded SLNs could be a potential new product for CE treatment.

## Introduction

Hydatid disease is a zoonotic disease caused by the larval stages of the *Echinococcusgranulosus*.^1-3^ The life cycle of this cestode involves two kinds of hosts: definitive hosts (dogs or wild carnivores) and intermediate hosts (humans and other mammals). This infection can progress to form a hydatid cyst that usually grows in the liver but is sometimes observed in other organs such as the lungs, spleen, abdominal cavity, and the nervous system.^4^

The cystic form of the cestode usually develops in the viscose of the intermediate host. Its diameter reaches several centimeters, and cysts contain hydatid fluid, protoscolices, and germinal cells.^5^ Hydatid disease infects almost 1% of all patients in general hospitals and has remained a health problem to this day.^6-9^

The infection has 1–3.6 million disability-adjusted life years worldwide, mainly in low-income countries. The rate of human infection to hydatid cysts is estimated to be 1.6−2.1 per 100 000 people.^8^ Of course, many reports mention the economic importance of hydatidosis in livestock.^7^

Because of the high prevalence of this zoonotic disease and the many health problems it causes, different methods for diagnosing and treating the disease are of considerable importance.^10,11^ Common approaches used to treat hydatid cysts in humans include surgery, drug therapy, and care/waiting procedures.^12^ Until the 1970s and the discovery of benzimidazoles, surgery was the only option. The first benzimidazole used for hydatid cyst treatment was metronidazole. ABZ was replaced due to its low absorption and some of its side effects.^13^

Today, ABZ is the most preferred drug for treating hydatid cysts. It is also used as a prophylaxis to prevent the formation of secondary cysts. The most important part of the drug is converted to three metabolites called albendazole sulfoxide (ABZSO), albendazole sulfone, and ABZ aminosulfone. The most effective of these metabolites is ABZSO.^14^ ABZ has little solubility in water, with an absorption rate of about 0.4% in a 400 mg oral unit.^15,16^

However, ABZ has its own disadvantages, such as its poor absorption and rapid metabolism. Thus, it seems that it cannot eliminate all of the protoscolex released after surgery. In addition, it has low permeability, meaning that its value in the plasma and cyst is lower than needed, while ABZ has a better anti cestode effect.^17-19^ As mentioned, ABZ has side effects, including toxic liver, leukopenia, thrombocytopenia, and hair loss. One way to increase the solubility of ABZ, thus increasing its therapeutic effect, is to ionize it in an acidic environment. However, this increase in solubility is insufficient for absorbing high concentrations of ABZ.^20^ Current nanotechnology methods have increased drug solubility and permeability. As a result, the therapeutic effect of drugs can be increased while side effects are decreased.^5^

This study aimed to evaluate the nephrotoxicity caused by ABZ and ABZ-loaded solid lipid nanoparticles (SLNs) in mice with experimental hydatid cysts.

## Material and Methods

### Sample collection and preparation

In this experimental study, samples of hydatid cysts were prepared from a slaughterhouse in Ahvaz, the capital city of Khuzestan province. The cysts were transported to the Department of Parasitology, Faculty of Medicine. Hydatid cysts were removed from the livers of the infected sheep under aseptic conditions and washed three times with phosphate-buffered saline (PBS). The cyst fluid of fertile hydatid cysts containing protoscolices was aspirated, and after washing three times with PBS, their fertility was tested using a microscopic examination for the presence of live protoscolices.^17,21^

### Preparation and synthesis of nanoparticles

SLNs, with and without the drug, were prepared using a high-pressure homogenizer and micro emulsion. In order to determine the percentage of drug-loaded and the pharmaceutical content of nanoparticles, two methods were used directly and indirectly. For the morphological evaluation of nanoparticles and determination of particle size, electron microscope imaging and scatteroscope were used.

### Preparation of ABZ loaded SLN

ABZ-loaded SLNs were provided by high-pressure homogenization and a modified micro-emulsification technique. Briefly, 7 g of comprisal 888 ATO (glyceryl dibehenate/behenate (Gattefosse, France) was added to a 250-mL beaker and then transferred to a boiling water bath at 75°C. Subsequently, 500 mg of ABZ (kindly donated by Damloran Razak Pharmaceutical Co., Ltd.) was slowly added to the melt, and the drug was fully dissolved in the lipid(s). In the next step, the hot aqueous-surfactant solution (containing 100 mL deionized water, 1 g Tween 80, and 1 g polyvinyl alcohol) previously heated at the same temperature under magnetic stirring was added gradually into the ABZ loaded lipid while stirring. The hot nano-emulsion was slowly dispersed in 100 mL of cold deionized water (4°C) and homogenized at 12 000 rpm for 30 minutes using an Ultra Turrax homogenizer (IKA®-Werke GmbH & Co., KG, Germany) to obtain a nanoparticle suspension. The suspension was further homogenized by a high-pressure homogenizer (AVESTIN, Canada) under a pressure of 500 bar and two homogenization cycles, followed by two times of centrifugation at 30 000 g for 30 minutes. In the presence of 5% sucrose (serving as a cryoprotectant), the sediments were lyophilized in a freeze-drier (Operon Co., Ltd., South Korea). The final products were stored in the refrigerator until needed. Free drug SLNs were prepared similarly.^22^

### Nanoparticles characterization

#### Particle size determination

The average particle size of SLNs was determined by photon correlation spectroscopy (PCS) using a scatteroscope (Qudix-South Korea). About 1 mL of each sample was diluted (1:100) with deionized water and analyzed. The span calculation is the most common way to express distribution width.

### Scanning electron microscopy experiments

SEM images of ABZ nanoparticles were obtained using a Leo 1455 VP SEM (Carl Zeiss, Germany). Briefly, 1 mg of freeze-dried ABZ-loaded SLNs were separately re-dispersed in 1 ml of deionized water under mechanical stirring and then sonicated for 15 minutes by a bath sonicator. Two microliters of the obtained suspension were finely spread on a coverslip and dried at ambient temperature. Once dry, the samples were coated with gold, and the nanoparticles’ morphologies were imaged by the SEM apparatus.

### Drug loading (DL %) and drug entrapment efficiency (EE %) measurement

We used direct and indirect methods to determine the entrapment efficiency and drug loading of ABZ. The drug dosage in the optimum ABZ-loaded SLNs was determined by UV-spectrophotometry at 296 nm for ABZ. The control SLNs were prepared in the same way but without the drug. The drug loading percentage of ABZ nanoparticles was calculated as indicated below:

$$DL\%=\frac{\left(Wdrug\;loaded\right)}{\left(Wdrug\;loaded+Wlipid\right)}\times 100$$

*W drug loaded*: Amount of the drug loaded

*W lipid*: Amount of lipid matrix

$$EE\%=\frac{\left(Winitial\;drug-Wfree\;drug\right)}{\left(Winitial\;drug\right)}\times 100$$

*W initial drug*: Total weight of the drug

*W free drug*: Weight of the free drug in the supernatant

### Physical stability experiments

ABZ-loaded SLNs physical stability was carried out by determining the average particle size, polydispersity index and drug loading percentage one month after preparing at refrigerator temperature.^23,24^

### Effect of nanoparticles in hydatidosis infected mice

Eighty BALB/C mice, 6−8 weeks old, weighing 20-25 g were injected intraperitoneally with 500 μL of prepared suspension, containing 2000 live protoscolices.^25^ Eight months after infecting the mice with hydatid cyst, they were divided into two groups of negative and positive controls. Each group included 4 sub-groups. Each subgroup in the positive and the negative control groups received 0.5 mL of deionized water as control, 200 mg/kg of ABZ free forms, 200 mg/kg ABZ loaded in lipid nanoparticles (ABZ-SLN) and 0.5 mL carbon tetrachloride (CCl_4_).

### Biochemical surveys

Following the ethical guidelines, all mice were sacrificed and their blood was taken and the serum was separated to measure kidney functional factors, such as blood urea nitrogen (BUN) and creatinine.^18^ Also, the malondialdehyde (MDA) agent as a product of lipid peroxidation and glutathione (GSH) as an antioxidant were evaluated.

### Histopathological

A small portion of the kidney tissues of the mice was isolated and fixed in 10% formalin, followed by dehydration and molding with paraffin, and then the sections obtained from the micrometer were stained with hematoxylin and eosin (H & E) staining. The histology study was performed using an optical microscope to evaluate histological changes occurring due to treatment with SLN a control for ABZ and ABZ-loaded SLNs. In the histopathologic examination, changes in the kidney tissue, including necrosis and hemorrhage were investigated.

## Results

### Nanoparticles’ average size and zeta potential

The mean particle size and zeta potential of optimum ABZ-loaded SLNs were approximately 80.9 ± 8.1. The polydispersity index (PDI) was calculated as 0.46 ± 0.01. The zeta potential was −34.4 ± 3.2 mV.

### Scanning electron microscopy investigation

**Figure 1:**
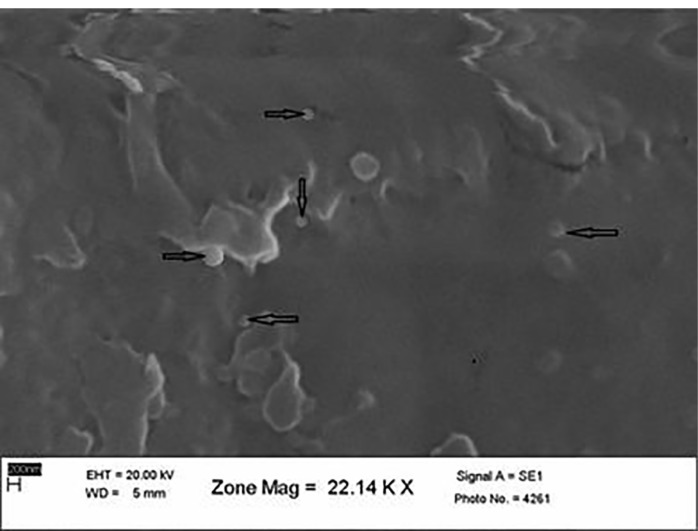
SEM Photography of the Optimum ABZ-loaded SLNs.

An scanning electron microscopy (SEM) investigation of the SLN formulations showed spherical particles smaller than 1 nm. Some particle aggregates were also observed. The size measured by PCS was confirmed with the images obtained through SEM. Particle aggregation might have been caused by the preparation of the re-dispersed freeze-dried sample, which then remained cryoprotectant during lyophilization. The SEM image of the optimum ABZ-loaded SLNs is shown in Figure 1.

### Physical stability

After one month of storage, no significant changes were observed in the average particle size, polydispersity index, or drug loading percentage of the optimum ABZ-loaded SLNs (Table 1).

**Table 1:** Stability profile of ABZ-loaded SLNs

**Time**	**Particle size**	**PDI**	**EE%**	**DL%**
First day	80.9±8.1	0.46±0.1	59.14	3.6
Week 4	81.1±9	0.46±0.2	59.02	3.57

### Drug encapsulation efficiency and drug loading

The entrapment efficiency (EE) and drug loading (DL) of the ABZ-loaded SLN formulation were 59.14%, 3.6%, respectively.

### BUN and creatinine factors

**Figure 2:**
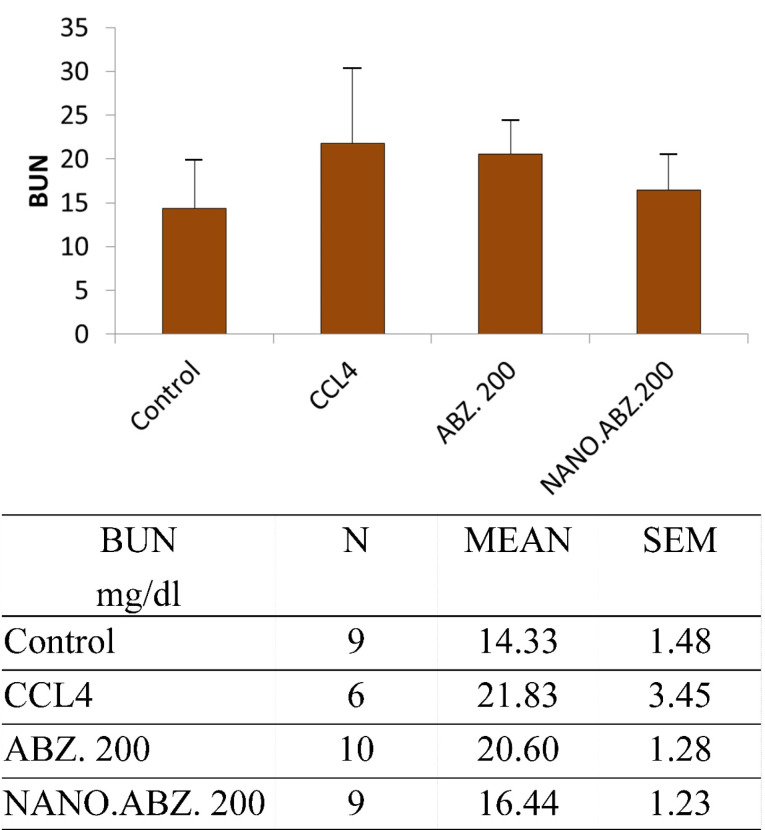
BUN value in mice with experimental hydatidosis.

The ABZ, ABZ-loaded SLNs, and the control group presented significantly different BUN levels (*P* = 0.008). There was also a significant difference between CCL4 and the negative control group (*P* = 0.042) (Figure 2).

Furthermore, in the experimental group without hydatid cysts, there was a significant difference between the ABZ and ABZ-loaded SLNs groups (*P* < 0.0006). There was no significant difference between CCL4 and control groups (*P* = 0.194).

**Figure 3:**
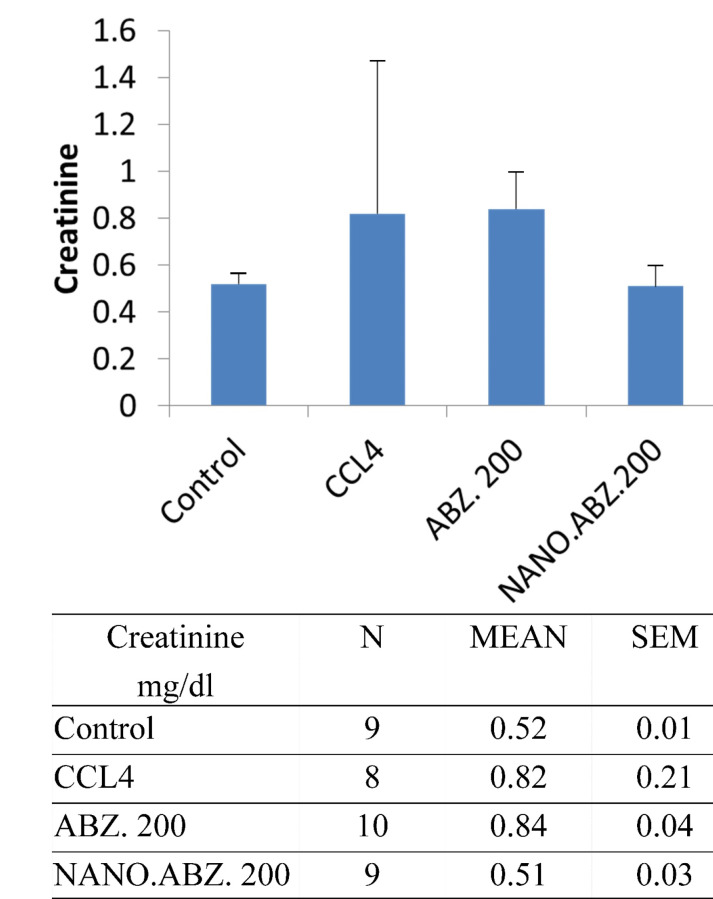
Creatinine values in mice with experimental hydatidosis.

In addition, the experimental group with hydatid cysts presented no significant difference between CCL_4_ and the control group (*P* = 0.981) regarding their creatinine levels. However, significant differences were detected between the ABZ, ABZ-loaded SLNs, and control groups (*P* <0.0001). Specifically, significant differences were observed between the control and ABZ groups and between the ABZ and ABZ-loaded SLNs groups (Figure 3). In the experimental group without hydatid cysts, no significant difference in creatinine value was observed between the CCL4 and control groups (*P* < 0.096). However, a significant difference arose between the ABZ, ABZ-loaded SLN, and control groups (*P* < 0.0003) (specifically, the difference was between the control and ABZ groups).

### Kidney malondialdehyde and glutathione factors

Our evaluation of MDA levels in the infected group indicated that the ABZ and ABZ-loaded SLN groups were not significantly different from the control group (*P* = 0.486). Also, MDA levels were reduced in the non-infected group. Regarding the GSH factor in infected and non-infected groups, the ABZ and ABZ-loaded SLN groups were not significantly different from the control group.

### Histopathological study

**Figure 4:**
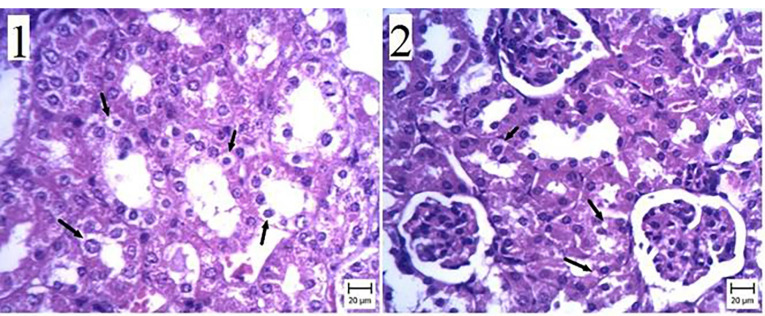
The kidney of the infected group. (1) Kidney infected with a hydatid cyst—the arrow indicates membranous glomerulonephritis. (2) Negative control group—the thin wall of the capillaries can be seen (Bar: 1 and 2: 20 μm). (H&E staining).

**Figure 5:**
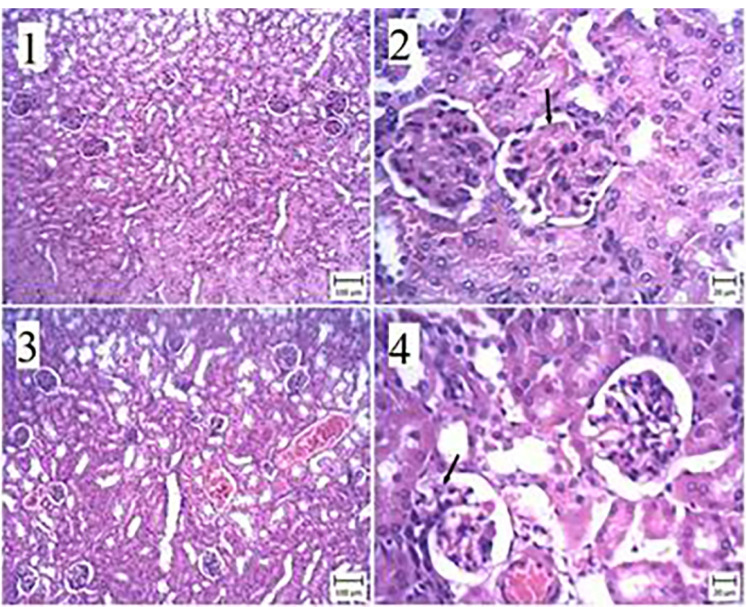
The kidney of a mouse. (1) and (2) The ABZ group with 200 + hydatid cyst—the membrane glomerulitis is characterized by an increase in pink matter inside the capillary sheath (arrow). (3) and (4) ABZ Group 200—the capillary wall inside the capillary sheath (arrow) is thin compared to Figure 2. (Bar: 1 and 3: 100 μm, 2 and 4: 20 μm). (H&E staining).

**Figure 6:**
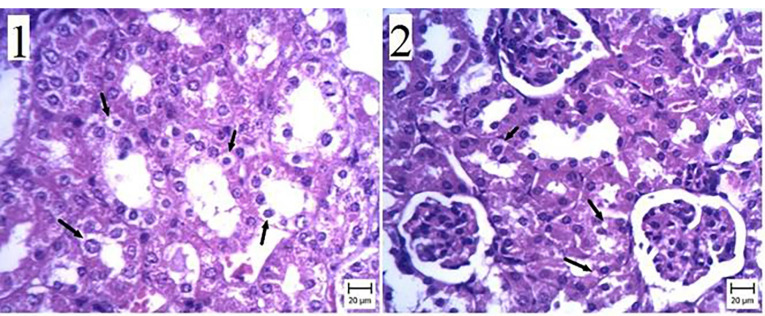
The kidney of a mouse. (1) Carbon tetrachloride + hydatid cyst group—note the severe degeneration of the lining of the urinary tract (arrow). (2) Carbon tetrachloride group—cell degeneration is indicated by the arrow. (Bar: 20 μm). (H&E staining).

We observed membranous glomerulonephritis in our histopathological investigation of the kidney of mice with experimental hydatid cysts. In some nephrons, the glomeruli were large, and pink substances were more prominent between cells (Figure 4). This alteration was seen in the nephropathy of the ABZ, ABZ-loaded SLN, and control groups (Figures 4 and 5). Some urinary tubule cells had also degenerated in the CCL4 group infected with the hydatid cyst. Conversely, no pathologic changes were seen in the non-infected group. All tubules, Bowman’s capsules, and interstitial tissues were normal (Figures 6).

## Discussion

To date, there is no alternative drug to ABZ for treating cystic echinococcosis (CE). Therefore, improving ABZ derivatives such as SLNs could improve treatment efficacy. Also, definitive treatment can be achieved by removing all cyst components through surgery. Even then, therapeutic procedures should be completed using protoscolocidal agents for an extended period before and after surgery. These methods should be safe and effective. ABZ is known to be effective against CE. Therefore, ABZ and mebendazole (MBZ) is the best drug-based treatment for cases when there are multiple small cysts in several organs.^12,17,26^

Nanoparticles play an important role in drug delivery systems because they deliver the drug at a specific time and with a controlled dose to the targeted tissue, resulting in faster and more specific treatment for the patient. In addition, these systems are safe carriers that can be easily manufactured on a large scale; they can also be used for the prolonged exposure and control of drug release.^27-29^

When using nano drugs, their biochemical and histopathological effects should be evaluated. Some studies have reported that ABZ-loaded SLNs have adequate efficacy for multiple aspects of hydatid cysts. More recently, ABZ-loaded SLNs were found to be effective in experimental hydatidosis.^17,22^ However, their side effects have not been investigated thoroughly. ABZ-loaded SLNs’ considerable effectiveness in experimental CE raised the demand for chemotherapeutic drugs when treating CE in humans, either as an alternative to surgery or to complement it.

In the current study, kidney functions were evaluated by measuring the serum levels of BUN, creatinine, MDA, and GSH, along with histopathological changes due to ABZ-loaded SLNs among BALB/c mice with experimental hydatid cyst.

Our results revealed considerable changes in the abovementioned kidney-related parameters (with significant differences observed between the ABZ and ABZ-loaded SLN groups). However, these changes were within the normal range. Therefore, it seems that the SLN preparation of ABZ is safe for humans. Nevertheless, these findings should be investigated in more detail before being applied to humans.

Regarding the histopathological changes induced by drug consumption, the experimental groups exhibited more cytotoxic effects than the control groups. However, these changes did not significantly affect kidney function. No similar information or findings were found in the literature.

Concerning the histopathological effects of drug consumption, membranous glomerulonephritis was observed in the kidneys of BALB/c experimental CE cases. Some nephrons presented larger glomeruli with more cells in between them. This alteration was seen in the nephropathy of the ABZ, ABZ-loaded SLN, and control groups.

The likely main causes of increased BUN levels are the decreased glomerular filtration rate, rapid cell destruction from infections, and increased catabolism. It seems that these histopathological alterations among the experimental groups do not affect kidney functions.

MDA is produced by the peroxidation of unsaturated fatty acids in the human body. It is active and highly reactive. Still, since MDA itself is an active and highly reactive compound by attacking other molecules, it binds covalently and strongly. Thus, it affects the function of other molecules and, ultimately, cell function. Our evaluation of MDA levels in the infected group showed no significant difference between ABZ and ABZ-loaded SLNs (*P* = 0.486). This finding indicates that oxidative stress does not change when this method is used.

GSH is a potent antioxidant that protects important cellular components from reacting with oxygenated functional groups such as free radicals and peroxides. A comparison between ABZ and ABZ-loaded SLNs revealed no significant differences in GSH factor. This result indicates the absence of tissue toxicity and tissue lesions, as well as the resuscitation of GSH. Oxidative injuries (and subsequent slight changes) occurred due to the toxic nature of CCl_4_.

## Conclusion

Experimental BALB/c hydatid cyst groups were treated with ABZ-loaded SLNs. We observed some post-treatment differences between the experimental and control groups regarding the histopathological changes in kidney-related and biochemical parameters based on serum levels. However, all kidney functions remained within the normal range. Therefore, it seems that the ABZ-loaded SLN derivative can be considered an applicable and safe drug for CE treatment after further assessment. However, before these findings are applied to human CE cases and clinical trials, their safety should be confirmed, and drug delivery values in the liver and lungs should be considered.

## Ethical Issues

This research was approved by the committee for ethics of Ahvaz University of Medical Sciences with the code (IR.AJUMS.ABHC.REC.1397.037).

## Conflict of Interest

There is no conflict of interest to declare.

## Acknowledgments

This article has been extracted from a Ph.D. thesis written by Kobra Kohansal in the Department of Parasitology, School of Medicine, Ahvaz Jundishapur University of Medical Sciences (Registration No. 330094237).
